# Penehyclidine for prevention of postoperative nausea and vomiting following bimaxillary orthognathic surgery: a randomized, double-blind, controlled trial

**DOI:** 10.1007/s00540-021-03017-4

**Published:** 2021-11-05

**Authors:** Li-Kuan Wang, Tong Cheng, Xu-Dong Yang, Guo-Li Xiong, Nan Li, Dong-Xin Wang

**Affiliations:** 1 Department of Anesthesiology and Critical Care Medicine, Peking University First Hospital, No.8 Xishiku street, Beijing, 100034 China; 2 Department of Anesthesiology, Peking University School and Hospital of Stomatology, Beijing, China; 3 Outcomes Research Consortium, Cleveland, OH USA

**Keywords:** Penehyclidine, Postoperative nausea and vomiting, Bimaxillary surgery, Orthognathic surgery

## Abstract

**Purpose:**

To investigate the efficacy and safety of low-dose bolus plus continuous infusion of penehyclidine in preventing postoperative nausea and vomiting (PONV) following bimaxillary surgery.

**Methods:**

Three hundred fifty-four patients were randomly allocated into three groups. In the Control group, placebo (normal saline) was injected before anesthesia and infused over 48 h after surgery; in the Bolus group, 0.5 mg penehyclidine was injected before anesthesia, whereas placebo was infused after surgery; in the Infusion group, 0.25 mg penehyclidine were injected before anesthesia, another 0.25 mg penehyclidine was infused after surgery. The primary endpoint was the incidence of PONV within 72 h.

**Results:**

A total of 353 patients were included in intention-to-treat analysis. The PONV incidence was 61.0% (72/118) in the Control group, 40.2% (47/117) in the Bolus group, and 28.0% (33/118) in the Infusion group. The incidence was significantly lower in the Bolus group than in the Control group (RR 0.66; 95% CI 0.51–0.86; adjusted *P* = 0.003) and in the Infusion group than in the Control group (RR 0.46; 95% CI 0.33–0.63; adjusted *P* < 0.001); the difference between the Infusion and Bolus groups was not statistically significant (RR 0.70; 95% CI 0.48–1.00; adjusted *P* = 0.144). Emergence agitation occurred more frequently in the Bolus group than in the Control group (36.8% [43/117] vs. 21.2% [25/118], adjusted *P* = 0.027), but did not differ significantly between the Infusion and Control groups.

**Conclusions:**

A low-dose bolus plus continuous infusion of penehyclidine was effective in preventing PONV without increasing emergence agitation.

**Trial registration:**

Clinicaltrials.gov. Identifier: NCT04454866.

**Supplementary Information:**

The online version contains supplementary material available at 10.1007/s00540-021-03017-4.

## Introduction

Postoperative nausea and vomiting (PONV) is one of the most frequent adverse complications after surgery and is strongly related to patients’ dissatisfaction [1]. It had reported that, compared with pain and decreased mental alertness, PONV was the most undesirable scenario during postoperative recovery [2].

Orthognathic surgery is usually performed for the correction of dentofacial deformities. PONV is extremely common after orthognathic surgery [3]. Early postoperative factors such as lip numbness, orofacial swelling, oral stimulation of the glossopharyngeal nerve, and swallowing blood all contribute to the development of PONV [4]. Compared with single-jaw surgery, bimaxillary surgery is followed by an even higher incidence of PONV despite antiemetic prophylaxis [4]. In a prospective cohort study, 72.2% of patients experienced nausea and 43.1% developed vomiting after bimaxillary surgery [5]. Occurrence of PONV may be life-threatening in bimaxillary patients; maxillomandibular elastic tractions, facial swelling and pain may all affect mouth-opening, increase the risk of aspiration and lead to asphyxia [6]. Therefore, it is important to prevent PONV in patients following bimaxillary surgery.

The pathophysiology of PONV is complex and involves various pathways and receptors [7]. No single drug used for preventing PONV is completely effective. It is recommended that combined antiemetics should be administered for preventing PONV in high-risk patients [8]. A 5-hydroxytryptamine 3 receptor antagonist plus dexamethasone is a recommended combination for PONV prophylaxis. However, even with combined therapy, the incidence of postoperative nausea was up to 50% in orthognathic patients and higher in bimaxillary cases [9]. Therefore, combined therapy with a third drug of another category is reasonable for preventing PONV in these patients.

It is realized that activation of the central cholinergic system, especially the M3 muscarinic acetylcholine receptor, plays an important role in the pathogenesis of PONV [10, 11]. Muscarinic antagonist such as scopolamine has been effectively used for PONV prevention [12]. Penehyclidine is a new muscarinic antagonist with high selectivity of the M3 receptor [13]. Previous studies showed that prophylactic penehyclidine administered before surgery helped to prevent PONV [14, 15]. Since the mean elimination half-life of a single-dose penehyclidine is about 10.35 h, whereas high frequent PONV may persist for up to 96 h after orthognathic surgery [16], we hypothesize that a low-dose bolus plus continuous infusion of penehyclidine may be more effective for PONV prevention. The purpose of this study is to investigate the efficacy and safety of single-dose plus continuously administrated penehyclidine in preventing PONV in patients undergoing bimaxillary surgery.

## Methods

### Study design

This was a double-blinded, randomized, controlled trial with three arms. The study protocol was approved by the Ethics Committee of Peking University Hospital of Stomatology (PKUSSIRB-202055076, 23 June 2020) and registered with ClinicalTrials.gov (NCT04454866, 29 June 2020). The study was conducted in Peking University Hospital of Stomatology (Beijing, China) in accordance with the CONSORT guidelines. Written informed consent was obtained from each participant.

### Participants

The inclusion criteria were adult patients aged ≥ 18 but < 60 y, with a body mass index ≥ 18 but < 30 kg/m^2^, scheduled to undergo elective bimaxillary orthognathic surgery, and required postoperative analgesia pump after surgery. Patients were excluded if they met any of the following: (1) presence of glaucoma; (2) allergic to penehyclidine, atropine, scopolamine, or other anticholinergic drugs; (3) acute or chronic nausea and/or vomiting, or gastrointestinal motility disorders before surgery; (4) received antiemetic therapy within 12 h before surgery; (5) history of schizophrenia, Parkinson's disease, profound dementia, or language barrier; or (6) severe hepatic dysfunction (Child–Pugh class C), severe renal dysfunction (requirement of renal replacement therapy before surgery), or American Society of Anesthesiologists (ASA) physical classification ≥ IV. We also excluded patients who participated in other clinical studies.

### Randomization and intervention

Random numbers were generated using the SPSS software in a 1:1:1 ratio and sealed in sequentially numbered opaque envelopes. The envelopes were opened before anesthesia by an anesthesia nurse who prepared the study drugs but did not participate in the rest of the study. The study drugs, placebo (normal saline) and/or penehyclidine hydrochloride (Chengdu Lisite Pharmaceutical Co., Ltd., Chengdu, China) for each patient in one of the three groups, were prepared with normal saline to the volume of 5 ml in two identical syringes. The envelops were closed again after study drug preparation until the end of the trial. Consequently, care providers, outcome assessors and patients were blinded to study group assignment.

Patients were randomly allocated into three groups. For patients in the Control group, a dose of placebo was injected intravenously before anesthesia induction; another dose of placebo was added to the intravenous analgesia pump. For patients in the Bolus group, a dose of 0.5 mg penehyclidine was injected intravenously before anesthesia induction; a dose of placebo was added to the intravenous analgesia pump. For patients in the Infusion group, a dose of 0.25 mg penehyclidine was injected intravenously before anesthesia induction; a dose of 0.25 mg penehyclidine was added to the intravenous analgesia pump. The intravenous analgesia pump, which was otherwise prepared with sufentanil (1.25–1.5 μg/kg) and tropisetron (10 mg) and diluted with normal saline to 100 ml, was provided for postoperative analgesia at a continuous infusion rate of 2 ml/h for 48 h.

### Anesthesia and perioperative care

No premedication was given. Intraoperative monitoring included electrocardiogram, noninvasive blood pressure, pulse oxygen saturation, end-tidal concentration of carbon dioxide, inhalational anesthetic concentration, and urine output.

Before anesthesia induction, all patients were given 10 mg dexamethasone; midazolam was administered intravenously at the discretion of attending anesthesiologists. General anesthesia was induced with sufentanil/remifentanil, propofol, and rocuronium/cis-atracurium. Nasotracheal intubation was performed. General anesthesia was maintained with intravenous infusion of propofol and remifentanil/sufentanil, with or without inhalational sevoflurane and/or nitrous oxide or dexmedetomidine infusion, at the discretion of attending anesthesiologists. Mechanical ventilation was established with a mixture of oxygen-air or oxygen-nitrous oxide. Vasoactive drugs were used to maintain hemodynamics stable or to induce intentional hypotension when clinically indicated. At the end of the surgery, nonsteroidal anti-inflammatory drugs (NSAIDs) and/or opioids were administered when considered necessary. For all patients, 2 mg tropisetron were given; the intravenous analgesia pump was attached and initiated.

After surgery, patients were transferred to the post-anesthesia care unit (PACU) with nasotracheal intubation. Dexmedetomidine sedation was provided, and NSAIDs and/or opioids were administered when considered necessary. Patients were extubated when they regained consciousness, fully recovered from paralysis, and had normal airway protective reflexes and circulatory status. The decision to transfer patients from PACU to general wards was decided by attending anesthesiologists but was usually in the next morning. Rescue antiemetics (metoclopramide and/or tropisetron) were prescribed by attending anesthesiologists or surgeons.

### Data collection and outcome assessment

Baseline data included demographic and morphometric characteristics, surgical diagnosis, preoperative comorbidities (and Charlson Comorbidity Index [17, 18]), history of smoking, drinking, motion sickness, and previous PONV (and Apfel’s score [19]), and results of relevant laboratory tests. Cognitive function was assessed with the Mini-Mental State Examination (MMSE; score ranges from 0 to 30, with a higher score indicating better function [20]). Sleep quality was assessed with the numeric rating scale (NRS; an 11-point scale where 0 indicates the worst sleep and 10 the best sleep). Pain severity was assessed with the NRS (an 11-point scale where 0 indicates no pain and 10 the worst pain). Delirium was assessed with the Confusion Assessment Methods (CAM) [21]. Intraoperative data included duration of anesthesia, types and doses of anesthetics and other medications, type and duration of surgery, and fluid balance.

Patients were evaluated for PONV at 6, 12, 24, 48, and 72 postoperative hours, and for delirium twice daily (8:00 to 10:00 and 18:00 to 20:00) during the first 5 postoperative days or until hospital discharge. Patients were then followed up 30 days after surgery via telephone communication. Nausea was diagnosed by direct questioning with severity assessed with the 11-point NRS ranging from 0 (no nausea) to 10 (the worst nausea). Vomiting was diagnosed when patients retched or expulsed intra-gastric contents. Delirium was assessed with the CAM or, for patients who remained intubated, the CAM for the Intensive Care Unit [22]. Investigators performing delirium assessment had been trained before initiating the trial [23]. Other postoperative complications were generally defined as newly occurred medical conditions that were harmful for patients’ recovery and required therapeutic intervention, i.e., grade II or higher on the Clavien-Dindo classification [24]. Cognitive function on the 30^th^ day after surgery was assessed with the modified Telephone Interview for Cognitive Status (TICS-m; score ranges from 0 to 50, with a higher score indicating better function) by verbal communication via telephone [25].

The primary endpoint was the incidence of PONV, defined as the development of any nausea, retching, or vomiting within 72 h after surgery. Secondary endpoints included: (1) incidence of PONV and moderate-to-severe nausea (NRS ≥ 4) during different time periods after surgery (0 to 6 h, > 6 to 12 h, > 12 to 24 h, > 24 to 48 h, and > 48 to 72 h); (2) incidence of moderate-to-severe nausea within 72 h after surgery; (3) severity of 72-h PONV. No PONV was defined as the absence of any nausea or emetic symptoms; mild PONV as the occurrence of mild nausea or one episode of vomiting; moderate PONV as moderate-to-severe nausea, or vomiting for 2 times or more, or any nausea that required only one rescue antiemetic therapy; severe PONV as more than two emetic episodes or necessitating more than one dose of rescue antiemetics [26]; (4) use of rescue antiemetics within 72 h after surgery; (5) incidence of postoperative delirium (POD) within the first 5 days after surgery; (6) length of stay in hospital after surgery; (7) incidence of other complications within 30 days after surgery; (8) all-cause 30-day mortality; and (9) cognitive function on the 30^th^ day after surgery. Other endpoints included: (1) the incidence of moderate-to-severe pain (a NRS pain score ≥ 4) at different time periods after surgery; (2) use of rescue analgesics within 72 h after surgery; and (3) subjective sleep quality within 3 days after surgery.

Adverse events were monitored for up to 72 h after surgery. Potential adverse events included dry mouth, fever (> 37.5 °C), dizziness, urinary retention (required urine re-catheterization), emergence agitation (Richmond Agitation-Sedation Scale ≥  + 2; score ranges from –5 [unarousable] to + 4 [combative] and 0 indicates alert and calm [27]), bradycardia (< 50 beat/min or a decrease of > 30% from baseline, and required therapy), tachycardia (> 100 beat/min or an increase of > 30% from baseline, and required therapy), hypotension (< 90 mm Hg or a decrease of > 30% from baseline, and required therapy), hypertension (> 180 mm Hg or an increase of > 30% from baseline, and required therapy), and desaturation (pulse oxygenation saturation < 90%).

## Statistical analysis

### Sample size estimation

In a retrospective study of our patients who underwent bimaxillary surgery from 1 April 2018 to 30 September 2019, the incidence of PONV was 46.2% and 66.7%, respectively, in patients with and without single-dose penehyclidine (Ethics Approval: PKUSSIRB-201947098; registered at clinicaltrials.gov, No. NCT04112771). A single-arm pilot study of our group (Ethics Approval: PKUSSIRB-201952180; registered at www. chictr.org.cn, No. ChiCTR2000028967) showed that PONV occurred in 23.3% of patients given penehyclidine infusion (a bolus injection of 0.25 mg before anesthesia induction, followed by a continuous infusion of 0.25 mg over 48 h) following bimaxillary surgery. With the significance level set at 0.05/3 = 0.0167 and power at 80%, the calculated sample size required to detect differences among the three groups was 112 patients in each group. Considering a drop-out rate of about 5%, we planned to include 118 patients in each group. Sample size calculation was performed using PASS 11.0 software (NCSS Statistical Software, East Kaysville, UT).

### Data analysis

Data analysis was performed on a modified intention-to-treat population; that is, all patients were analyzed in the group to which they were randomized, excluding those who withdraw consents before intervention. For the primary outcome, analysis was also performed in the per-protocol population, excluding patients who dropped out of the trial.

For baseline, intraoperative and postoperative data, quantitative data were compared with analysis of variance or Kruskal–Wallis test; qualitative data were compared with chi-squared test or Fisher’s exact test.

Our primary endpoint, the incidence of PONV within 72 h, was compared with chi-squared test, with differences between groups expressed as relative risk (RR) and 95% CI. Similar analyses were performed for the per-protocol population. Post-hoc exploratory analyses were performed to assess heterogeneity of the primary outcome in predefined sub-groups including age, female sex, motion sickness/PONV history, smoking history, use of nitrous oxide, use of sevoflurane, and duration of surgery. Treatment-by-covariate interactions were adjusted for sub-group factors using logistic regression.

For secondary endpoints, quantitative data were compared with analysis of variance or Kruskal–Wallis test. Qualitative data were compared with chi-squared test or Fisher’s exact test. Repeatedly measured variables (NRS of sleep quality) were compared with the general linear model. Missing data were not replaced. The difference between groups was quantified as the RR or median difference (MD) and 95% CI.

For post hoc pairwise comparison, *P* values were adjusted with the Bonferroni method. A two-side *P* < 0.05 was regarded statistically significant. Statistical analysis was performed with the SPSS 21.0 software package (IBM SPSS Inc., Chicago, IL, USA).

## Results

### Patient recruitment and characteristics

**Fig. 1:**
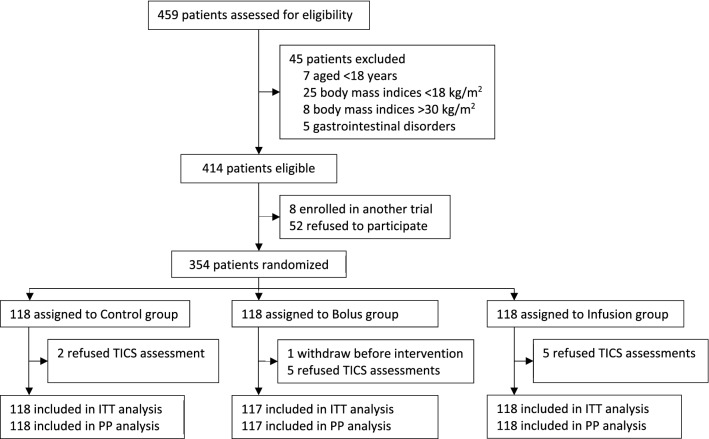
Flowchart of the study

From 7 July 2020 to 15 March 2021, 459 patients scheduled for elective bimaxillary orthognathic surgery were assessed for eligibility. Of these, 414 patients were eligible; 354 patients were recruited and randomized into three groups, with 118 patients in each group. During the study period, one patient in the Bolus group withdraw consent before intervention; the remaining patients were included in the modified intention-to-treat population (Fig. 1). The three groups were well balanced regarding the baseline data (Table 1). Intraoperative and postoperative data were also comparable among the three groups (Table 2).

**Table 1:** Baseline data

	Control group (*n* = 118)	Bolus group (*n* = 117)	Infusion group (*n* = 118)	*P* value
Age (y)	25 (22, 28)	24 (21, 28)	25 (22, 29)	0.560
Female sex	81 (68.6%)	82 (70.1%)	81 (68.6%)	0.963
Body mass index (kg/m^2^)	20.0 (18.8, 22.7)	20.3 (18.8, 22.0)	21.1 (19.2, 23.3)	0.168
Education (y)^a^	16 (15, 17)	16 (15, 16)	16 (15, 16)	0.166
Preoperative comorbidities				
Asthma	1 (0.8%)	2 (1.7%)	6 (5.1%)	0.133
Obstructive sleep apnea^b^	10 (8.5%)	4 (3.4%)	5 (4.2%)	0.182
Hyperthyroidism	2 (1.7%)	0 (0%)	0 (0%)	0.331
Hypothyroidism	1 (0.8%)	0 (0%)	1 (0.8%)	> 0.999
Chronic gastritis	0 (0%)	2 (1.7%)	0 (0%)	0.109
Hepatitis B	2 (1.7%)	4 (3.4%)	0 (0%)	0.091
Depression^c^	2 (1.7%)	1 (0.9%)	2 (1.7%)	> 0.999
Anxiety^c^	2 (1.7%)	1 (0.9%)	0 (0%)	0.552
Allergic rhinitis	2 (1.7%)	4 (3.4%)	2 (1.7%)	0.609
Epilepsy	1 (0.8%)	0 (0%)	0 (0%)	> 0.999
Gout	0 (0%)	1 (0.9%)	0 (0%)	0.331
Hypertension	2 (1.7%)	0 (0%)	0 (0%)	0.331
Lung cancer	1 (0.8%)	0 (0%)	0 (0%)	> 0.999
Charlson comorbidity index	0 (0, 0)	0 (0, 0)	0 (0, 0)	0.150
Previous surgery	37 (31.4%)	33 (28.2%)	41 (34.7%)	0.558
Drinking history^d^	2 (1.7%)	1 (0.9%)	3 (2.5%)	0.874
No smoking history^e^	108 (91.5%)	111 (94.9%)	105 (89.0%)	0.257
Motion sickness/PONV history	14 (11.9%)	18 (15.4%)	24 (20.3%)	0.201
Apfel risk factors				
1	6 (5.1%)	3 (2.6%)	9 (7.6%)	0.358
2	30 (25.4%)	32 (27.4%)	28 (23.7%)	
3	71 (60.2%)	66 (56.4%)	61 (51.7%)	
4	11 (9.3%)	16 (13.7%)	20 (16.9%)	
ASA classification				
I	87 (73.7%)	97 (82.9%)	91 (77.1%)	0.230
II	31 (26.3%)	20 (17.1%)	27 (22.9%)	
Laboratory tests				
Hemoglobin (g/L)	139 (128, 152)	136 (127, 151)	136 (129, 147)	0.926
Alanine aminotransferase (IU/L)	12 (9, 19)	13 (9, 20)	12 (9, 17)	0.366
Aspartate aminotransferase (IU/L)	16 (14, 19)	17 (15, 20)	17 (14, 19)	0.525
Albumin (g/L)	47.1 (44.9, 48.5)	46.8 (45.1, 48.8)	46.8 (45.4, 48.3)	0.803
Na^+^ (mmol/l)	141.8 (140.7, 142.8)	141.5 (140.6, 142.7)	141.9 (141.0, 142.8)	0.350
K^+^ (mmol/l)	4.18 ± 0.30	4.13 ± 0.34	4.17 ± 0.29	0.484
Glucose (mmol/l)	4.8 (4.7, 5.1)	4.9 (4.6, 5.1)	4.9 (4.6, 5.2)	0.433
Creatinine (μmol/l)	56.2 (49.8, 68.6)	57.9 (51, 66.9)	58.9 (50.8, 70.9)	0.377
Mini-Mental State Examination (score)^f^	30 (29, 30)	30 (29, 30)	30 (29, 30)	0.178
Delirium	0 (0%)	0 (0%)	0 (0%)	> 0.999
NRS of sleep quality (point)^g^	7 (6, 9)	7 (6, 9)	7 (6, 9)	0.773
NRS of preoperative pain (point)^h^	0 (0, 0)	0 (0, 0)	0 (0, 0)	0.798
Preoperative chronic pain^i^	6 (5.1%)	8 (6.8%)	7 (5.9%)	0.851
Fasting time (h)	9.0 (7.0, 11.0)	9.0 (7.0, 11.0)	10.0 (7.0, 11.0)	0.155
Surgery schedule				
Morning (8:00–12:00)	56 (47.5%)	57 (48.7%)	63 (53.4%)	0.878
Afternoon (12:00–18:00)	60 (50.8%)	57 (48.7%)	53 (44.9%)	
Night (after 18:00)	2 (1.7%)	3 (2.6%)	2 (1.7%)	

Data are mean ± SD, median (IQR), or *n* (%)

*ASA* American society of anesthesiologists, *NRS* numeric rating scale, *PONV* postoperative nausea and vomiting

^a^From elementary school

^b^Diagnosed with polysomnography

^c^Diagnosed by psychiatrists

^d^Daily consumption of the equivalent of 80 g of alcohol for at least 1 year

^e^Smoking history was defined as those who have smoked more than 100 cigarettes in their lifetime

^f^Score ranges from 0 to 30, with a higher score indicating better function

^g^Assessed with the 11-point NRS ranging from 0 (the worst sleep) to 10 (the best sleep)

^h^Assessed with the 11-point NRS ranging from 0 (none pain) to 10 (the worst pain)

^i^Refers to the chronic pain in the temporomandibular joint and/or masticatory muscle at rest or movement that affected daily life activities including mood, mouth opening, mastication or speaking, as judged by patients themselves

**Table 2:** Intra- and postoperative data

	Control group (*n* = 118)	Bolus group (*n* = 117)	Infusion group (*n* = 118)	*P* value
Intraoperative data				
Prophylactic dexamethasone^a^	118 (100.0%)	117 (100.0%)	118 (100.0%)	–
Duration of anesthesia (min)	246 (209, 301)	263 (225, 300)	249 (222, 292)	0.570
Intraoperative medications				
Nitrous oxide	49 (41.5%)	46 (39.3%)	53 (44.9%)	0.681
Sevoflurane	100 (84.7%)	102 (87.2%)	101 (85.6%)	0.863
Midazolam	83 (70.3%)	86 (73.5%)	77 (65.3%)	0.381
Dose of midazolam (mg)	2.0 (0, 2.5)	2.0 (0, 2.5)	2.0 (0, 2.5)	0.929
Propofol	118 (100%)	117 (100%)	118 (100%)	–
Dose of propofol (mg/kg)	21.7 (14.8, 27.6)	23.8 (16.4, 31.3)	21.5 (15.3, 27.7)	0.110
Sufentanil	117 (99.2%)	117 (100%)	118 (100%)	0.368
Dose of sufentanil (μg/kg)	0.6 (0.4, 0.8)	0.6 (0.5, 0.7)	0.6 (0.4, 0.7)	> 0.999
Remifentanil	118 (100%)	117 (100%)	118 (100%)	–
Dose of remifentanil (μg/kg)	30 (21, 42)	33 (25, 43)	32 (24, 41)	0.203
Dezocine	51 (43.2%)	53 (45.3%)	55 (46.6%)	0.870
Rocuronium	33 (28.0%)	40 (34.2%)	35 (29.7%)	0.564
Cis-atracurium	87 (73.7%)	77 (65.8%)	83 (70.3%)	0.414
Dexmedetomidine	70 (59.3%)	78 (66.7%)	79 (66.9%)	0.383
Dose of dexmedetomidine (μg/kg)	0.3 (0, 1.4)	0.4 (0, 1.2)	0.4 (0, 1.2)	0.834
Non-steroidal anti-inflammatory drugs	85 (72.0%)	86 (73.5%)	84 (71.2%)	0.923
Flurbiprofen axetil	85 (72.0%)	85 (72.6%)	83 (70.3%)	0.920
Ketorolac	0 (0%)	1 (0.9%)	1 (0.8%)	0.776
Antihypertensive drugs	55 (46.6%)	50 (42.7%)	46 (39.0%)	0.496
Nicardipine	47 (39.8%)	41 (35.0%)	39 (33.1%)	0.537
Esmolol	39 (33.1%)	34 (29.1%)	28 (23.7%)	0.283
Urapidil	2 (1.7%)	4 (3.4%)	7 (5.9)	0.224
Vasopressors	16 (13.6%)	14 (12.0%)	18 (15.3%)	0.763
Ephedrine	8 (6.8%)	8 (6.8%)	9 (7.6%)	0.961
Methoxamine	10 (8.5%)	8 (6.8%)	9 (7.6%)	0.894
Atropine	2 (1.7%)	2 (1.7%)	2 (1.7%)	> 0.999
Prophylactic tropisetron^b^	118 (100.0%)	117 (100.0%)	118 (100.0%)	–
Duration of surgery (min)	195 (165, 244)	208 (176, 245)	199 (174, 240)	0.482
Additional procedures	105 (89.0%)	107 (91.5%)	105 (89.0%)	0.771
Genioplasty	96 (81.4%)	97 (82.9%)	94 (79.7%)	0.816
Iliac bone harvest	3 (2.5%)	4 (3.4%)	2 (1.7%)	0.651
Extractions	40 (33.9%)	44 (37.6%)	43 (36.4%)	0.832
Intravenous fluid (ml)	1600 (1600, 2100)	2100 (1600, 2100)	1600 (1600, 2100)	0.576
Infusion of hydroxyethyl starch	52 (44.1%)	57 (48.7%)	53 (44.9%)	0.748
Estimated blood loss (ml)	250 (200, 300)	250 (200, 300)	255 (200, 300)	0.815
Urine output (ml)	400 (200, 685)	500 (205, 750)	400 (250, 700)	0.542
Postoperative data				
Duration in PACU (h)	15 (12, 18)	15 (12, 18)	15 (11, 19)	0.919
Time to extubation (min)	65 (45, 110)	70 (40, 110)	65 (44, 110)	0.973
Use of dexmedetomidine in PACU	100 (84.7%)	99 (84.6%)	95 (80.5%)	0.612
Intravenous fluid in PACU (ml)	1800 (1675, 2050)	1800 (1800, 1975)	1900 (1800, 2300)	0.411
Urine output in PACU (ml)	1075 (650, 1600)	1200 (700, 1700)	1300 (800, 1863)	0.114
Drainage in PACU (ml)	80 (49, 120)	70 (40, 118)	70 (40, 120)	0.652
Medication during 72 h after surgery				
Tropisetron in analgesia pump^c^	118 (100.0%)	117 (100.0%)	118 (100.0%)	–
Total sufentanil equivalent dose (μg)^d^	80 (70, 90)	80 (75, 90)	83 (75, 95)	0.550
Non-steroidal anti-inflammatory drugs	29 (24.6%)	33 (28.2%)	25 (21.2%)	0.459
Flurbiprofen axetil	19 (16.1%)	23 (19.7%)	18 (15.3%)	0.635
Loxoprofen	18 (15.3%)	15 (12.8%)	10 (8.5%)	0.272
Celecoxib	0 (0%)	2 (1.7%)	0 (0%)	0.109

Data are median (IQR) or *n* (%)

*PACU* post-anesthesia care unit

^a^Dexamethasone 10 mg administered before anesthesia induction

^b^Tropisetron 2 mg administered before the end of surgery

^c^Tropisetron 10 mg added to the analgesia pump and infused over a 48-h period

^d^Sufentanil equivalent dose consumed within 72 h after surgery (including postoperative analgesia pump)

### Efficacy outcomes

**Fig. 2:**
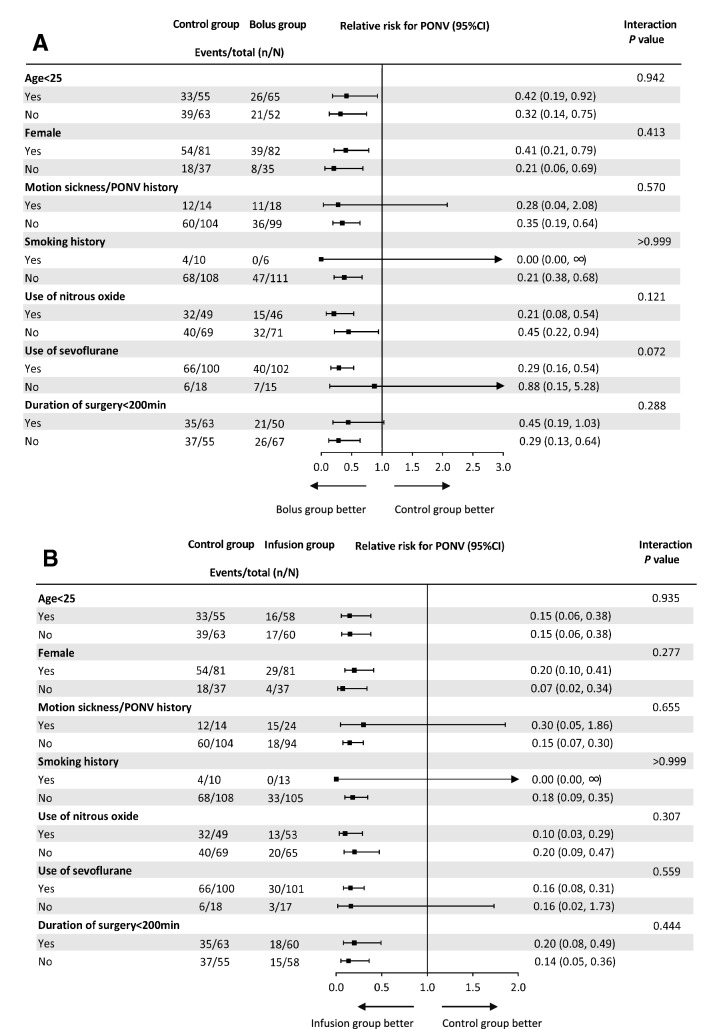
Forest plot in predefined subgroups. Forest plot assessing the effect of Bolus group vs. Control group (**A**) and the effect of Infusion group vs. Control group (**B**) in predefined subgroups. Logistic models were applied for the assessment of treatment-by-covariate interactions. Treatment-by-covariate interactions were adjusted for each subgroup factor, including age, female sex, motion sickness/PONV history, smoking history, use of nitrous oxide, use of sevoflurane, and duration of surgery. PONV, postoperative nausea and vomiting

PONV occurred in 61.0% (72/118) of patients in the Control group, 40.2% (47/117) in the Bolus group, and 28.0% (33/118) in the Infusion group within 72 h. The incidence of PONV within 72 h was significantly lower in the Bolus group than in the Control group (RR 0.66; 95% CI 0.51 to 0.86; adjusted *P* = 0.003) and in the Infusion group than in the Control group (RR 0.46; 95% CI 0.33 to 0.63; adjusted *P* < 0.001). The difference between the Infusion and Bolus groups was not statistically significant (RR 0.70; 95% CI 0.48 to 1.00; adjusted *P* = 0.144) (Table 3). Per-protocol analysis gave the same results. There were no significant interactions between penehyclidine administration and predefined factors (Fig. 2A, B).

**Table 3:** Efficacy outcomes

	Control group (*n* = 118)	Bolus group (*n* = 117)	Infusion group (*n* = 118)	Bolus vs. control		Infusion vs. control		Infusion vs. Bolus	
				RR or MD (95% CI)	Adjusted *P* value ^a^	RR or MD (95% CI)	Adjusted *P* value ^a^	RR or MD (95% CI)	Adjusted *P* value ^a^
Primary outcome									
PONV within 72 h	72 (61.0%)	47 (40.2%)	33 (28.0%)	0.66 (0.51, 0.86)	**0.003**	0.46 (0.33, 0.63)	** < 0.001**	0.70 (0.48, 1.00)	0.144
Secondary outcomes									
Moderate-to-severe nausea within 72 h^b^	49 (41.5%)	31 (26.5%)	26 (22.0%)	0.64 (0.44, 0.92)	**0.045**	0.53 (0.36, 0.79)	**0.003**	0.83 (0.53, 1.31)	> 0.999
Severity of PONV									
None	46 (39.0%)	70 (59.8%)	85 (72.0%)	–	**0.021**	–	** < 0.001**	–	0.432
Mild	20 (16.9%)	16 (13.7%)	7 (5.9%)	–		–		–	
Moderate	34 (28.8%)	24 (20.5%)	20 (16.9%)	–		–		–	
Severe	18 (15.3%)	7 (6.0%)	6 (5.1%)	–		–		–	
Rescue antiemetics within 72 h	35 (29.7%)	15 (12.8%)	15 (12.7%)	0.43 (0.25, 0.75)	**0.006**	0.43 (0.25, 0.74)	**0.003**	0.99 (0.51, 1.94)	> 0.999
Metoclopramide	31 (26.3%)	14 (12.0%)	10 (8.5%)	0.46 (0.26, 0.81)	**0.015**	0.32 (0.17, 0.63)	** < 0.001**	0.71 (0.33, 1.53)	> 0.999
Tropisetron^c^	10 (8.5%)	2 (1.7%)	8 (6.8%)	0.20 (0.05, 0.90)	0.054	0.80 (0.33, 1.96)	> 0.999	3.97 (0.86, 18.28)	0.306
5-day delirium	3 (2.5%)	5 (4.3%)	3 (2.5%)	1.68 (0.41, 6.87)	> 0.999	1.00 (0.21, 4.85)	> 0.999	0.60 (0.15, 2.43)	> 0.999
LOS in hospital (d)	5 (5, 6)	5 (4, 6)	5 (5, 6)	MD = 0 (0, 0)	> 0.999	MD = 0 (0, 0)	> 0.999	MD = 0 (0, 0)	> 0.999
30-day complications	9 (7.6%)	5 (4.3%)	5 (4.2%)	0.56 (0.19, 1.62)	0.831	0.56 (0.19, 1.61)	0.810	0.99 (0.30, 3.34)	> 0.999
Blood transfusion	1 (0.8%)	0 (0%)	1 (0.8%)	–	> 0.999	1.00 (0.06, 15.80)	> 0.999	–	> 0.999
Wound infection	4 (3.4%)	3 (2.6%)	4 (3.4%)	0.76 (0.17, 3.31)	> 0.999	1.00 (0.26, 3.91)	> 0.999	1.32 (0.30, 5.78)	> 0.999
Reoperation	4 (3.4%)	2 (1.7%)	0 (0%)	0.50 (0.09, 2.70)	> 0.999	–	0.366	–	0.741
30-day mortality	0 (0%)	0 (0%)	0 (0%)	–	–	–	–	–	–
TICS-m at 30 days (score)	37 (36, 39) [2]	37 (36, 39) [5]	38 (36, 39) [5]	MD = 0 (1, 0)	> 0.999	MD = 0 (1, 0)	0.681	MD = 0 (1, 0)	> 0.999
Other outcomes									
Rescue analgesics within 72 h	44 (37.3%)	42 (35.9%)	36 (30.5%)	0.96 (0.69, 1.35)	> 0.999	0.82 (0.57, 1.17)	0.813	0.85 (0.59, 1.22)	> 0.999

Data are *n* (%) or median (IQR). Adjusted *P* values in bold indicate those < 0.05 after Bonferroni correction. Numbers in square brackets indicate patients refused TICS assessment

*RR* relative risk, *MD* median difference, *NRS* numerical rating scale, *PONV* postoperative nausea and vomiting, *LOS* length of stay, *TICS*-m telephone interview for cognitive status- modified

^a^The *P* value was adjusted according to the Bonferroni method

^b^Defined as NRS nausea score of ≥ 4

^c^Except tropisetron in postoperative analgesia pump

**Fig. 3:**
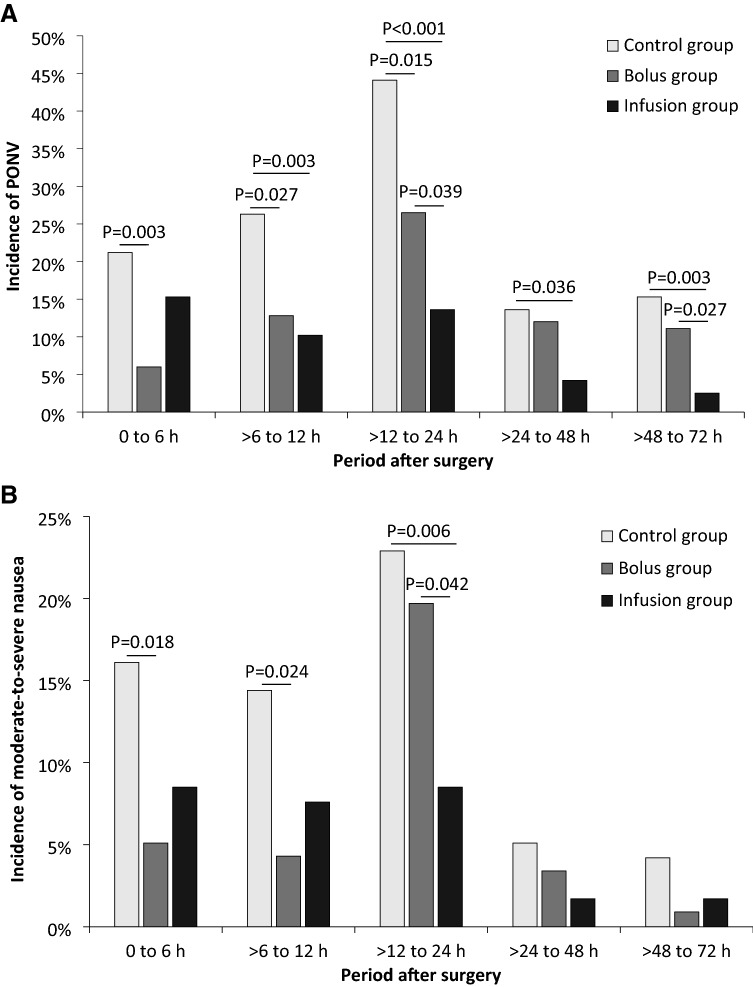
Incidences of PONV (**A**) and moderate-to-severe nausea (**B**) during different time periods after surgery. When compared with the Control group, the incidence of PONV was significantly lower in the Bolus group from 0 to 6 h, > 6 to 12 h, and > 12 to 24 h after surgery, and was significantly lower in the Infusion group from > 6 to 12 h, > 12 to 24 h, > 24 to 48 h, and > 48 to 72 h after surgery; when compared with the Bolus group, it was significantly lower in the Infusion group from > 12 to 24 h and > 48 to 72 h after surgery (**A**). When compared with the Control group, the incidence of moderate-to-severe nausea was significantly lower in the Bolus group from 0 to 6 h and > 6 to 12 h after surgery; when compared with the Bolus group, it was significantly lower in the Infusion group from > 12 to 24 h after surgery (**B**). *P* values were adjusted with Bonferroni method. PONV, postoperative nausea and vomiting. Please also see Table A1.

Regarding the incidence of PONV during different time periods, when compared with the Control group, it was significantly lower in the Bolus group from 0 to 6 h, > 6 to 12 h, and > 12 to 24 h after surgery, and was significant lower in the Infusion group from > 6 to 12 h, > 12 to 24 h, > 24 to 48 h, and > 48 to 72 h after surgery; when compared with the Bolus group, it was significantly lower in the Infusion group from > 12 to 24 h and > 48 to 72 h after surgery (Fig. 3A and Table A1).

Regarding the incidence of moderate-to-severe nausea during different time periods, when compared with the Control group, it was significantly lower in the Bolus group from 0 to 6 h and > 6 to 12 h after surgery; when compared with the Bolus group, it was significantly lower in the Infusion group from > 12 to 24 h after surgery (Fig. 3B and Table A1). The total incidence of moderate-to-severe nausea within 72 h was significantly lower in the Bolus and Infusion groups than in the Control group (RR 0.64; 95% CI 0.44 to 0.92; adjusted *P* = 0.045 and RR 0.53; 95% CI 0.36 to 0.79; adjusted *P* = 0.003, respectively) (Table 3).

The severity of PONV within 72 h showed a statistically significant difference among the three groups. It was less severe in the Bolus and Infusion groups than in the Control group (adjusted *P* = 0.021 and *P* < 0.001, respectively). The requirements of rescue antiemetics within 72 h were also significantly less in the Bolus and Infusion groups than in the Control group (RR 0.43; 95% CI 0.25 to 0.75; adjusted *P* = 0.006 and RR 0.43; 95% CI 0.25 to 0.74; adjusted *P* = 0.003, respectively), mainly due to less metoclopramide consumption (Table 3).

Other secondary outcomes, including delirium within 5 days, length of stay in hospital after surgery, complications within 30 days, and cognitive score assessed with TICS-m at 30 days were comparable among the three groups. No patient died during the study period (Table 3 and Table A1).

### Safety outcomes

The proportion of patients who complained of dry mouth was significantly higher in the Bolus and Infusion groups than in the Control group but was significantly lower in the Infusion group than in the Bolus group. The incidence of emergence agitation was significantly higher in the Bolus group than in the Control group, but there was no significant difference between the Infusion and the Control groups. The occurrence of other adverse events did not differ among the three groups (Table 4).

**Table 4:** Safety outcomes

	Control group (*n* = 118)	Bolus group (*n* = 117)	Infusion group (*n* = 118)	*P* value
Total incidence	87 (73.7%)	99 (84.6%)	93 (78.8%)	0.122
Dry mouth	27 (22.9%)	73 (62.4%)*	54 (45.8%)*†	** < 0.001**
Fever^a^	72 (61.0%)	61 (52.1%)	69 (58.5%)	0.367
Dizziness	10 (8.5%)	18 (15.4%)	11 (9.3%)	0.183
Urinary retention^b^	1 (0.8%)	3 (2.6%)	0 (0.0%)	0.132
Emergence agitation^c^	25 (21.2%)	43 (36.8%)*	31 (26.3%)	**0.026**
Bradycardia^d^	4 (3.4%)	6 (5.1%)	6 (5.1%)	0.765
Tachycardia^e^	1 (0.8%)	2 (1.7%)	3 (2.5%)	0.707
Hypotension^f^	2 (1.7%)	2 (1.7%)	1 (0.8%)	0.874
Desaturation^g^	2 (1.7%)	0 (0.0%)	0 (0.0%)	0.331
Diarrhea ^h^	5 (4.2%)	1 (0.9%)	4 (3.4%)	0.360

Data are *n* (%). *P* values in bold indicate those of < 0.05

^*^Adjusted *P* < 0.05 compared with control group

^†^Adjusted *P* < 0.05 compared with penehyclidine bolus group. The *P* values were adjusted for multiple comparisons based on the Bonferroni method

^a^Body temperature > 37.5 °C

^b^Required urine re-catheterization

^c^Defined as Richmond Agitation-Sedation Scale (score ranges from –5 [unarousable] to + 4 [combative] and 0 indicates alert and calm) ≥  + 2

^d^Defined as heart rate < 50 beat min^−1^ or a decrease of > 30% from baseline, and required therapeutic interventions

^e^Defined as heart rate > 100 beat min^−1^ or an increase of > 30% from baseline, and required therapeutic interventions

^f^Defined as systolic blood pressure < 90 mm Hg or a decrease of > 30% from baseline, and required therapeutic interventions

^g^Pulse oxygenation saturation < 90%

^h^Diarrhea required therapeutic interventions

## Discussion

Our results showed that, despite multimodal prophylaxis, the incidence of PONV remained high following bimaxillary orthognathic surgery. Patients given a single bolus of penehyclidine at anesthesia induction had less PONV but more emergence agitation; those given a low-dose bolus followed by a continuous infusion of penehyclidine had less PONV without a significant increase in emergence agitation.

Two prior studies investigated the effect of penehyclidine in preventing PONV. Zhang et al. [14] compared effects of tropisetron, penehyclidine, or a combination of tropisetron and penehyclidine in 120 women scheduled for gynecological laparoscopic surgery. Penehyclidine (0.01 mg/kg, maximal dose 1.0 mg) was injected intramuscularly 20–40 min before anesthesia. They reported an incidence of vomiting of 30% with tropisetron, 45% with penehyclidine, and 10% with combined tropisetron and penehyclidine, respectively (*P* < 0.05). The combined therapy was thus more effective in preventing PONV than either tropisetron or penehyclidine alone. In a recent randomized trial of 228 pediatric patients who had strabismus surgery, penehyclidine (0.01 mg/kg, maximal dose 0.5 mg) or placebo was injected intravenously immediately after anesthesia induction. The results showed that patients given penehyclidine had a significantly lower incidence of PONV within 48 h (30.7% vs. 54.8%; *P* < 0.01) [15].

In the above studies, a single-dose penehyclidine was administered for PONV prevention. In accord with its elimination half-life, the antiemetic effect of penehyclidine is diminished or disappeared after 24 h [14, 15]. However, PONV following orthognathic surgery may persist for up to 96 h [16]. We, therefore, tested the hypothesis that a bolus plus infusion administration of penehyclidine could be more effective in reducing PONV. In the present study, 61.0% of patients in the Control group experienced PONV within 72 h following bimaxillary surgery; this was in line with previous studies in a similar patient population [5, 28]. Compared with the Control group, patients given a single bolus penehyclidine (Bolus group) had a 34% lower incidence of PONV within 72 h, close to the effect reported by Sun and colleagues [15]. As expected, patients given bolus-plus-infusion penehyclidine (Infusion group) had a 72-h PONV incidence 30% even lower than those in the Bolus group, although not statistically significant. We note that the incidence of PONV from > 12 to 24 h and from > 48 to 72 h as well as the incidence of moderate-to-severe nausea from > 12 to 24 h were all significantly lower in the Infusion group than in the Bolus group. These results indicated that low-dose bolus plus infusion administration of penehyclidine was more effective in preventing PONV in our patients. We did not find any significant sub-group differences, indicating that the effects of penehyclidine administration on PONV apply broadly.

A major concern in using anticholinergic drugs is cognitive side effects [29]. Perioperative administration of anticholinergic drugs are important risk factors of POD [30]. In a recent meta-analysis including 33 randomized controlled trials and 4017 patients, use of penehyclidine significantly increased POD when compared with atropine [31]. However, cognitive side effects were not assessed in the above studies investigating the effects of penehyclidine in preventing PONV [14, 15]. In our results, the incidence of POD was low (total 3.1%) and did not differ among the three groups. This could be explained by the fact that our patients were young (median age 24 years) and healthy (77.9% in ASA class I), because we excluded those at high risk of delirium [32, 33]; the doses of penehyclidine used were relatively small (total 0.5 mg), whereas the cognitive side effects are dose-dependent [31]; and a high rate of dexmedetomidine use in our patients might also have reduced delirium [34, 35].

Although POD was not increased, the incidence of emergence agitation was significantly higher in the Bolus group but not in the Infusion group. Emergence agitation is a common complication after oral and maxillofacial surgery [36, 37], and anticholinergic agents are a known risk factor [38]. Among other penehyclidine-related side effects, dry mouth is the most frequent one because the M3 receptors are distributed in the salivary gland [39]. In the present study, patients in both penehyclidine groups had more dry mouth than those given placebo, but dry mouth occurred less frequently in the Infusion group than in the Bolus group. Therefore, safety outcomes also support the use of low-dose bolus plus infusion strategy for PONV prevention.

Another anticholinergic agent that is frequently used for PONV prevention is scopolamine [40]. However, even low-dose transdermal scopolamine can produce significant effects on autonomic cardiovascular regulation as it increases vagal cardiac inhabitation and decrease blood pressure in healthy young subjects [41]. It is also reported that the use of transdermal scopolamine caused postoperative tachycardia [42]. As a selective M1 and M3 receptor antagonist, penehyclidine can inhibit vagal reflex and has little effects on heart rate [39]. Our results also showed that penehyclidine in both groups did not increase cardiovascular side effects. Lack of cardiovascular side effects might be an advantage of penehyclidine when used for PONV prevention.

In the present study, anesthesia was performed according to routine practice. As a result, all patients were given propofol for anesthesia induction and maintenance, and a high proportion was given supplemental inhalational anesthetics. From the point of view of PONV prevention, use of inhalational anesthetics is suboptimal [8]. However, inhalational anesthesia remains common for orthognathic surgery in many centers; one important reason is that volatile anesthetics are preferred to induce hypotension which is frequently required to reduce intraoperative bleeding [43–45]. Current evidence that the use of inhalational anesthetics is associated with increased risk of PONV mainly comes from studies comparing propofol anesthesia *versus* inhalational anesthesia [46, 47]. Whereas in our patients, those who were given inhalational anesthetics actually received combined intravenous-volatile anesthesia. In a recent meta-analysis, the risk of PONV in the recovery room was significantly reduced after combined intravenous-volatile anesthesia when compared with inhalational anesthesia, and no significant difference was found when compared with total intravenous anesthesia [48]. This also explains why a high proportion of our patients were given inhalational anesthetics during propofol maintenance. Furthermore, the use of nitrous oxide or sevoflurane inhalation was well balanced among the three groups of our patients.

There are several other limitations to the present study that merit discussion. First, we enrolled patients undergoing bimaxillary surgery, a patient population at very high risk of PONV. This limited the generalizability of our results. Second, all patients in our trial were given dexamethasone before anesthesia induction and tropisetron during the first 2 postoperative days. Therefore, our results could not reveal the effect of penehyclidine monotherapy in preventing PONV in bimaxillary patients. This is understandable because it is inappropriate to use monotherapy for PONV prevention in these high-risk patients [8].

## Conclusions

PONV remained common following bimaxillary surgery despite multimodal prophylaxis. A single bolus of penehyclidine in addition to antiemetics of other classes was effective in preventing PONV but was associated with increased emergence agitation; whereas a low-dose bolus plus a continuous infusion of penehyclidine was even more effective in preventing PONV without a significant increase in emergence agitation. Anesthesiologists should consider the low-dose bolus plus infusion regimen of penehyclidine administration for PONV prevention in high-risk patients.

## Supplementary Information

Below is the link to the electronic supplementary material.

Supplementary file1 (DOCX 26 KB)
