# Supplementary material for: Penehyclidine for prevention of postoperative nausea and vomiting following bimaxillary orthognathic surgery: a randomized, double-blind, controlled trial
Source: J Anesth. 2021 Nov 5;36(1):122–36. doi: 10.1007/s00540-021-03017-4 (PMC8807454; doi:10.1007/s00540-021-03017-4)
Supplement: Supplementary file 1 — Supplementary file1 (DOCX 26 KB) [file 540_2021_3017_MOESM1_ESM.docx]

Table A1. Secondary and other outcomes.

|  | **Control group (n=118)** | **Bolus group (n=117)** | **Infusion group (n=118)** | **Bolus vs. Control** | | **Infusion vs. Control** | | **Infusion vs. Bolus** | |
| --- | --- | --- | --- | --- | --- | --- | --- | --- | --- |
|  |  |  |  | **RR (95% CI) or Mean D±SE** | **Adjusted**  ***P* value ^a^** | **RR (95% CI) or Mean D±SE** | **Adjusted**  ***P* value ^a^** | **RR (95% CI) or Mean D±SE** | **Adjusted**  ***P* value ^a^** |
| **Secondary outcomes** |  |  |  |  |  |  |  |  |  |
| PONV |  |  |  |  |  |  |  |  |  |
| 0 to 6 h | 25 (21.2%) | 7 (6.0%) | 18 (15.3%) | 0.28 (0.13, 0.63) | **0.003** | 0.72 (0.42, 1.25) | 0.714 | 2.55 (1.11, 5.88) | 0.063 |
| >6 to 12 h | 31 (26.3%) | 15 (12.8%) | 12 (10.2%) | 0.49 (0.28, 0.86) | **0.027** | 0.39 (0.21, 0.72) | **0.003** | 0.79 (0.39, 1.62) | >0.999 |
| >12 to 24 h | 52 (44.1%) | 31 (26.5%) | 16 (13.6%) | 0.60 (0.42, 0.87) | **0.015** | 0.31 (0.19, 0.51) | **<0.001** | 0.51 (0.30, 0.88) | **0.039** |
| >24 to 48 h | 16 (13.6%) | 14 (12.0%) | 5 (4.2%) | 0.88 (0.45, 1.73) | >0.999 | 0.31 (0.12, 0.83) | **0.036** | 0.35 (0.13, 0.95) | 0.090 |
| >48 to 72 h | 18 (15.3%) | 13 (11.1%) | 3 (2.5%) | 0.73 (0.37, 1.42) | >0.999 | 0.17 (0.05, 0.55) | **0.003** | 0.23 (0.07, 0.78) | **0.027** |
| Moderate-to-severe nausea ^b^ |  |  |  |  |  |  |  |  |  |
| 0 to 6 h | 19 (16.1%) | 6 (5.1%) | 10 (8.5%) | 0.32 (0.13, 0.77) | **0.018** | 0.53 (0.26, 1.08) | 0.222 | 1.65 (0.62, 4.40) | 0.927 |
| >6 to 12 h | 17 (14.4%) | 5 (4.3%) | 9 (7.6%) | 0.30 (0.11, 0.78) | **0.024** | 0.53 (0.25, 1.14) | 0.288 | 1.79 (0.62, 5.17) | 0.831 |
| >12 to 24 h | 27 (22.9%) | 23 (19.7%) | 10 (8.5%) | 0.86 (0.52, 1.41) | >0.999 | 0.37 (0.19, 0.73) | **0.006** | 0.43 (0.22, 0.87) | **0.042** |
| >24 to 48 h | 6 (5.1%) | 4 (3.4%) | 2 (1.7%) | 0.67 (0.20, 2.32) | >0.999 | 0.33 (0.07, 1.62) | 0.843 | 0.50 (0.09, 2.66) | >0.999 |
| >48 to 72 h | 5 (4.2%) | 1 (0.9%) | 2 (1.7%) | 0.20 (0.02, 1.70) | 0.639 | 0.40 (0.08, 2.02) | >0.999 | 1.98 (0.18, 21.57) | >0.999 |
| **Other outcomes** |  |  |  |  |  |  |  |  |  |
| Moderate-to-severe pain ^b^ |  |  |  |  |  |  |  |  |  |
| 0 to 6 h | 27 (22.9%) | 22 (18.8%) | 27 (22.9%) | 0.82 (0.50, 1.36) | >0.999 | 1.00 (0.63, 1.60) | >0.999 | 1.22 (0.74, 2.01) | >0.999 |
| >6 to 12 h | 22 (18.6%) | 19 (16.2%) | 18 (15.3%) | 0.87 (0.50, 1.52) | >0.999 | 0.82 (0.46, 1.44) | >0.999 | 0.94 (0.52, 1.70) | >0.999 |
| >12 to 24 h | 25 (21.2%) | 31 (26.5%) | 19 (16.1%) | 1.25 (0.79, 1.98) | >0.999 | 0.76 (0.44, 1.30) | 0.948 | 0.61 (0.37, 1.01) | 0.156 |
| >24 to 48 h | 20 (16.9%) | 14 (12.0%) | 12 (10.2%) | 0.71 (0.38, 1.33) | 0.834 | 0.60 (0.30, 1.17) | 0.384 | 0.85 (0.41, 1.76) | >0.999 |
| >48 to 72 h | 9 (7.6%) | 8 (6.8%) | 5 (4.2%) | 0.90 (0.36, 2.24) | >0.999 | 0.56 (0.19, 1.61) | 0.810 | 0.62 (0.21, 1.84) | >0.999 |
| NRS of sleep quality |  |  |  |  |  |  |  |  |  |
| Day of surgery | 4 (2, 5) | 4 (3, 5) | 5 (3, 6) | 0±0 | >0.999 | -1±0 | 0.093 | 0±0 | 0.435 |
| Postoperative day 1 | 6 (5, 7) | 6 (5, 7) | 6 (5, 8) | 0±0 | >0.999 | 0±0 | 0.296 | 0±0 | 0.339 |
| Postoperative day 2 | 7 (6, 8) | 7 (6, 8) | 7 (6, 9) | 0±0 | 0.339 | 0±0 | 0.150 | 0±0 | >0.999 |
| Postoperative day 3 | 7 (6, 9) | 8 (6, 8) | 8 (6, 9) | 0±0 | >0.999 | 0±0 | 0.279 | 0±0 | >0.999 |

Data are n (%). Adjusted *P* values in bold indicate those <0.05 after Bonferroni correction.

PONV, postoperative nausea and vomiting; NRS, numeric rating scale.

^a^ The *P* value was adjusted according to the Bonferroni method.

^b^ Defined as NRS nausea score of ≥4.
